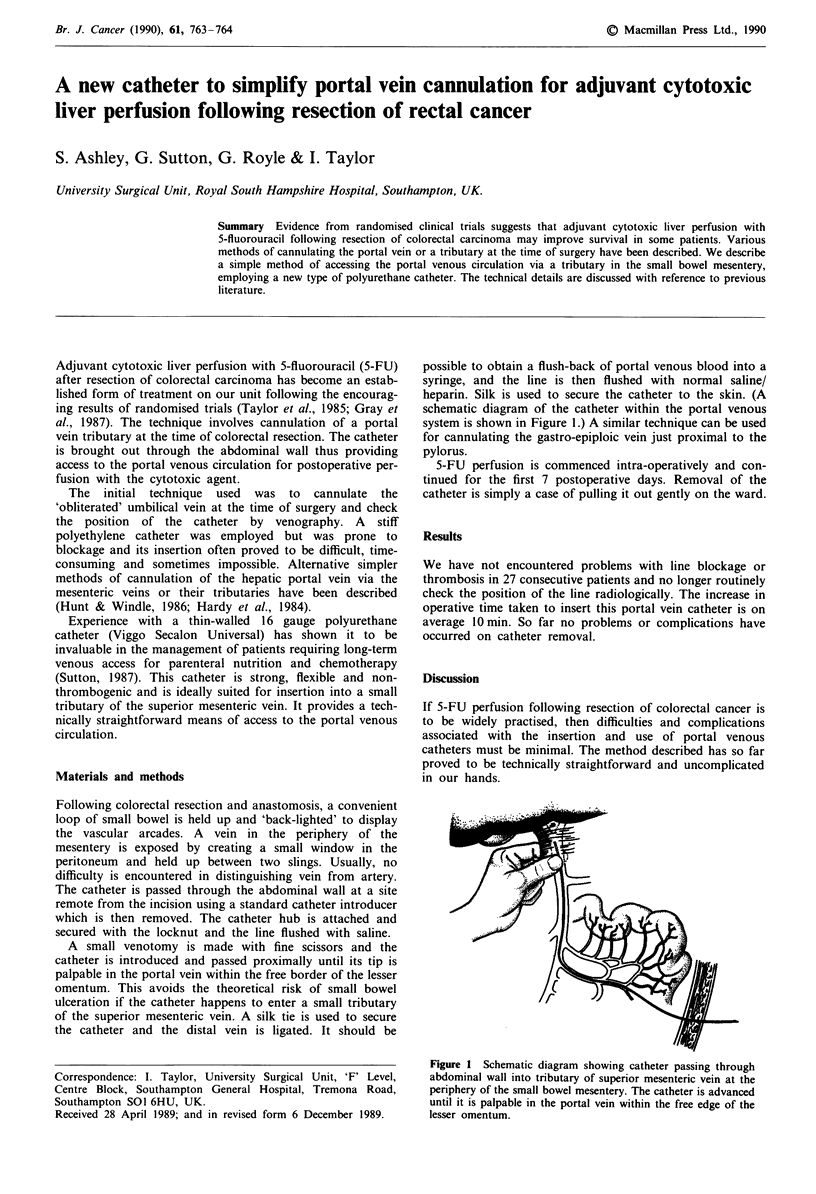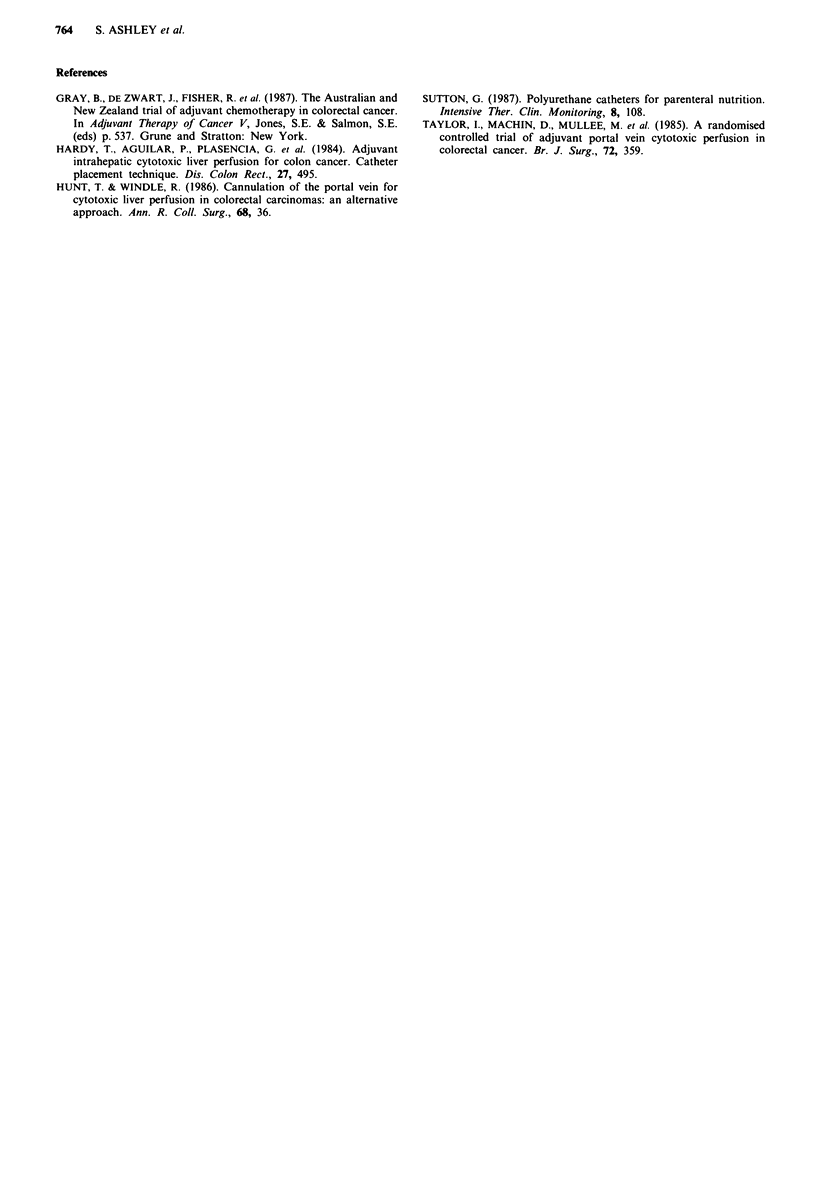# A new catheter to simplify portal vein cannulation for adjuvant cytotoxic liver perfusion following resection of rectal cancer.

**DOI:** 10.1038/bjc.1990.170

**Published:** 1990-05

**Authors:** S. Ashley, G. Sutton, G. Royle, I. Taylor

**Affiliations:** University Surgical Unit, Royal South Hampshire Hospital, Southampton, UK.

## Abstract

Evidence from randomised clinical trials suggests that adjuvant cytotoxic liver perfusion with 5-fluorouracil following resection of colorectal carcinoma may improve survival in some patients. Various methods of cannulating the portal vein or a tributary at the time of surgery have been described. We describe a simple method of accessing the portal venous circulation via a tributary in the small bowel mesentery, employing a new type of polyurethane catheter. The technical details are discussed with reference to previous literature.


*Br. J. Cancer* (1990), **61**, 763–764 © Macmillan Press Ltd., 1990

### A new catheter to simplify portal vein cannulation for adjuvant cytotoxic liver perfusion following resection of rectal cancer

S. Ashley, G. Sutton, G. Royle & I. Taylor

*University Surgical Unit, Royal South Hampshire Hospital, Southampton, UK.*

**Summary** Evidence from randomised clinical trials suggests that adjuvant cytotoxic liver perfusion with 5-fluorouracil following resection of colorectal carcinoma may improve survival in some patients. Various methods of cannulating the portal vein or a tributary at the time of surgery have been described. We describe a simple method of accessing the portal venous circulation via a tributary in the small bowel mesentery, employing a new type of polyurethane catheter. The technical details are discussed with reference to previous literature.

Adjuvant cytotoxic liver perfusion with 5-fluorouracil (5-FU) after resection of colorectal carcinoma has become an established form of treatment on our unit following the encouraging results of randomised trials (Taylor *et al.*, 1985; Gray *et al.*, 1987). The technique involves cannulation of a portal vein tributary at the time of colorectal resection. The catheter is brought out through the abdominal wall thus providing access to the portal venous circulation for postoperative perfusion with the cytotoxic agent.

The initial technique used was to cannulate the 'obliterated' umbilical vein at the time of surgery and check the position of the catheter by venography. A stiff polyethylene catheter was employed but was prone to blockage and its insertion often proved to be difficult, time-consuming and sometimes impossible. Alternative simpler methods of cannulation of the hepatic portal vein via the mesenteric veins or their tributaries have been described (Hunt & Windle, 1986; Hardy *et al.*, 1984).

Experience with a thin-walled 16 gauge polyurethane catheter (Viggo Secalon Universal) has shown it to be invaluable in the management of patients requiring long-term venous access for parenteral nutrition and chemotherapy (Sutton, 1987). This catheter is strong, flexible and non-thrombogenic and is ideally suited for insertion into a small tributary of the superior mesenteric vein. It provides a technically straightforward means of access to the portal venous circulation.

### Materials and methods

Following colorectal resection and anastomosis, a convenient loop of small bowel is held up and 'back-lighted' to display the vascular arcades. A vein in the periphery of the mesentery is exposed by creating a small window in the peritoneum and held up between two slings. Usually, no difficulty is encountered in distinguishing vein from artery. The catheter is passed through the abdominal wall at a site remote from the incision using a standard catheter introducer which is then removed. The catheter hub is attached and secured with the locknut and the line flushed with saline.

A small venotomy is made with fine scissors and the catheter is introduced and passed proximally until its tip is palpable in the portal vein within the free border of the lesser omentum. This avoids the theoretical risk of small bowel ulceration if the catheter happens to enter a small tributary of the superior mesenteric vein. A silk tie is used to secure the catheter and the distal vein is ligated. It should be possible to obtain a flush-back of portal venous blood into a syringe, and the line is then flushed with normal saline/heparin. Silk is used to secure the catheter to the skin. (A schematic diagram of the catheter within the portal venous system is shown in Figure 1.) A similar technique can be used for cannulating the gastro-epiploic vein just proximal to the pylorus.

5-FU perfusion is commenced intra-operatively and continued for the first 7 postoperative days. Removal of the catheter is simply a case of pulling it out gently on the ward.

### Results

We have not encountered problems with line blockage or thrombosis in 27 consecutive patients and no longer routinely check the position of the line radiologically. The increase in operative time taken to insert this portal vein catheter is on average 10 min. So far no problems or complications have occurred on catheter removal.

### Discussion

If 5-FU perfusion following resection of colorectal cancer is to be widely practised, then difficulties and complications associated with the insertion and use of portal venous catheters must be minimal. The method described has so far proved to be technically straightforward and uncomplicated in our hands.

**Figure 1** Schematic diagram showing catheter passing through abdominal wall into tributary of superior mesenteric vein at the periphery of the small bowel mesentery. The catheter is advanced until it is palpable in the portal vein within the free edge of the lesser omentum.

Correspondence: I. Taylor, University Surgical Unit, 'F' Level, Centre Block, Southampton General Hospital, Tremona Road, Southampton SO1 6HU, UK.
Received 28 April 1989; and in revised form 6 December 1989.

### References

GRAY, B., DE ZWART, J., FISHER, R. *et al.* (1987). The Australian and New Zealand trial of adjuvant chemotherapy in colorectal cancer. In *Adjuvant Therapy of Cancer V*, Jones, S.E. & Salmon, S.E. (eds) p. 537. Grune and Stratton: New York.

HARDY, T., AGUILAR, P., PLASENCIA, G. *et al.* (1984). Adjuvant intrahepatic cytotoxic liver perfusion for colon cancer. Catheter placement technique. *Dis. Colon Rect.*, **27**, 495.

HUNT, T. & WINDLE, R. (1986). Cannulation of the portal vein for cytotoxic liver perfusion in colorectal carcinomas: an alternative approach. *Ann. R. Coll. Surg.*, **68**, 36.

SUTTON, G. (1987). Polyurethane catheters for parenteral nutrition. *Intensive Ther. Clin. Monitoring*, **8**, 108.

TAYLOR, I., MACHIN, D., MULLEE, M. *et al.* (1985). A randomised controlled trial of adjuvant portal vein cytotoxic perfusion in colorectal cancer. *Br. J. Surg.*, **72**, 359.